# A non-targeted metabolomics analysis identifies wound-induced oxylipins in *Physcomitrium patens*

**DOI:** 10.3389/fpls.2022.1085915

**Published:** 2023-01-10

**Authors:** Hanno Christoph Resemann, Kirstin Feussner, Ellen Hornung, Ivo Feussner

**Affiliations:** ^1^ Department of Plant Biochemistry, Albrecht-von-Haller-Institute for Plant Sciences, University of Goettingen, Goettingen, Germany; ^2^ Service Unit for Metabolomics and Lipidomics, Goettingen Center for Molecular Biosciences (GZMB), University of Goettingen, Goettingen, Germany; ^3^ Department of Plant Biochemistry, Goettingen Center for Molecular Biosciences (GZMB), University of Goettingen, Goettingen, Germany

**Keywords:** bryophyte, cyclopentenones, lipid peroxidation, metabolite fingerprinting, octadecanoids, *Physcomitrella patens*, polyunsaturated fatty acids

## Abstract

Plant oxylipins are a class of lipid-derived signaling molecules being involved in the regulation of various biotic and abiotic stress responses. A major class of oxylipins are the circular derivatives to which 12-oxo-phytodienoic acid (OPDA) and its metabolite jasmonic acid (JA) belong. While OPDA and its shorter chain homologue *dinor*-OPDA (dnOPDA) seem to be ubiquitously found in land plants ranging from bryophytes to angiosperms, the occurrence of JA and its derivatives is still under discussion. The bryophyte *Physcomitrium patens* has received increased scientific interest as a non-vascular plant model organism over the last decade. Therefore, we followed the metabolism upon wounding by metabolite fingerprinting with the aim to identify jasmonates as well as novel oxylipins in *P. patens*. A non-targeted metabolomics approach was used to reconstruct the metabolic pathways for the synthesis of oxylipins, derived from roughanic, linoleic, α-linolenic, and arachidonic acid in wild type, the oxylipin-deficient mutants of *Ppaos1* and *Ppaos2*, the mutants of *Ppdes* being deficient in all fatty acids harboring a Δ^6^-double bond and the C20-fatty acid-deficient mutants of *Ppelo*. Beside of OPDA, *iso*-OPDA, dnOPDA, and *iso*-dnOPDA, three additional C18-compounds and a metabolite being isobaric to JA were identified to accumulate after wounding. These findings can now serve as foundation for future research in determining, which compound(s) will serve as native ligand(s) for the oxylipin-receptor COI1 in *P. patens*.

## Introduction

*Physcomitrium* (formerly *Physcomitrella*) *patens* is a non-vascular plant model organism that has received increased scientific interest over the last decade. Its genome was sequenced in 2008 (Rensing et al., 2008) and it is accessible for creating knock-out mutants *via* homologous recombination (Schaefer et al., 1991; Girke et al., 1998) as well as by clustered regularly interspaced short palindromic repeat (CRISPR)-CRISPR associated protein 9 (Cas9) mediated mutagenesis (Lopez-Obando et al., 2016; Collonnier et al., 2017). As a moss it is part of the paraphyletic group of bryophytes, which also includes the non-vascular plant divisions hornworts and liverworts with the model organism *Marchantia polymorpha* (Naramoto et al., 2021). It is assumed that the common evolutionary origin of the non-vascular bryophytes and the vascular tracheophytes lies relatively close to an event about 450 million years ago, when plants developed the ability to live on land (Clarke et al., 2011). This makes *P. patens* particularly interesting for studying similarities and differences in metabolic pathways between bryophytes and vascular plants with regard to the needed adaptation processes to life outside the water (Resemann et al., 2019).

Identifying novel metabolites that are involved in stress responses can be difficult because of the immense variety of compounds that are present in a living cell, especially in plants. Non-targeted metabolic fingerprinting analysis enables us to narrow our field of view on those components that actually show interesting patterns related to experimental conditions, may it be stress, growth conditions or genetic backgrounds (Dunn et al., 2013). Isolating these biomarkers from large data sets can then be followed up by identifying the exact chemical structure and function of the metabolite in question (Wishart, 2008). With the development of a variety of useful tools for metabolomics in plants (Roessner et al., 2001; Farag et al., 2012; Kaever et al., 2015; Feussner and Feussner, 2020; Feussner et al., 2022) we are now capable of identifying candidates for the more extensive analysis tests with higher confidence. Even if the exact function and chemical structure of an interesting biomarker cannot be elucidated in one, it can serve as the basis for further analyses in the future.

Land plants are immobile organisms and therefore have to deal with many different abiotic and biotic stresses that they cannot escape from. Oxylipins, a class of metabolites formed through the oxidation of unsaturated fatty acids (UFA), are in plants involved in processes regarding the response to these stresses (Wasternack and Feussner, 2018). They can be converted spontaneously or *via* specific enzymes into a plethora of compounds with varying degrees of oxidation and carbon-chain length. One important branch of the oxylipin biosynthesis is the formation of jasmonic acid (JA), a circular oxylipin deriving from the fatty acid α-linolenic acid (α-LeA, 18:3*n*-3), a polyunsaturated fatty acid (PUFA) (Wasternack and Song, 2017). Lipoxygenases (LOX) catalyze the formation of hydroperoxides from PUFAs. *Via* an allene oxide synthase (AOS), the hydroperoxide is further converted into an instable allene oxide, which spontaneously hydrolyses into ketols or the enzyme allene oxide cyclase (AOC) further catalyzes the formation of one precursor molecule for JA, 12-oxo phytodienoic acid (OPDA). Follow-up reactions include the enzyme OPDA-reductase isoform 3 (OPR3) and several steps of β-oxidation, which ultimately leads to the formation of JA. Its amino acid conjugate JA isoleucine (JA-Ile) is an important signal compound formed in tissues damaged by wounding of flowering plants (Howe et al., 2018). The wound response can be induced *via* feeding of insects like caterpillars or abiotic stresses like heavy rain or hail. In the laboratory the wounding is caused by damaging the leaves with forceps or razor blades (Farmer and Ryan, 1992). Besides wounding stress, JA and its precursor molecules in the biosynthetic pathway are furthermore associated with regulation of developmental processes, like root growth and pollen formation (Wasternack and Feussner, 2018).

In *P. patens*, several of the enzymes involved in the JA-biosynthesis are present, most notably seven active variants of LOX (Anterola et al., 2009), two and three variants each for AOS, and AOC, respectively (Bandara et al., 2009; Stumpe et al., 2010; Hashimoto et al., 2011; Neumann et al., 2012; Scholz et al., 2012). However, the detection of JA itself and its derivates is still under discussion. While two groups report on the accumulation of JA at very different levels in *P. patens* (Oliver et al., 2009; Bandara et al., 2009), we were so far not able to detect JA (Stumpe et al., 2010). However, it is still open whether the amounts of JA are below detection limit or JA is absent from this organism. In the other model bryophyte *M. polymorpha* the occurrence of JA and JA-Ile was again reported (Monte et al., 2018). While the absolute amount of OPDA after wounding in *M. polymorpha* was in the same range as measured for wounded leaves of *A. thaliana* (about 1 - 6 nmol/g fresh weight), JA showed only residual amounts in *M. polymorpha* (about 2 pmol/g fresh weight) and JA-Ile could not be detected. Indeed, recent studies concerning the COI1 receptor, which in flowering plants interacts with the active JA-derivate JA-Ile, revealed that the receptor itself appears early in plant evolution (Monte et al., 2018; Monte et al., 2019) and in both bryophytes the JA-precursor OPDA is detectable (Monte et al., 2018). Treating either bryophyte with OPDA inhibits plant growth, similar to how JA and JA-Ile affect growth in the vascular plant *A. thaliana* (Dathe et al., 1981; Staswick et al., 1992). Apparently, the COI1 receptor in *A. thaliana* has evolved to accept a different ligand, JA-Ile, than in *M. polymorpha*, which is assumed to be an OPDA-derived C-16 compound, *dinor*-OPDA (dnOPDA) (Monte et al., 2018). However, in *P. patens* it is not known if there is a similar compound fulfilling the function of JA-Ile in *A. thaliana*.

This study therefore aims to contribute to the discussion by identifying novel wounding-induced oxylipins in *P. patens*. For this purpose, we studied a knock-out (KO)-mutant of the enzyme PpAOS1, that was described before (Scholz et al., 2012). It is incapable of producing OPDA, and furthermore a KO-mutant of PpAOS2 with unknown association for wounding stress. *Via* analysis of a dataset obtained by non-targeted UPLC-HRMS analyses we reconstructed the oxylipin pathways in *P. patens* and we were able to identify yet unknown compounds that are absent in the *Ppaos1* mutant, while *Ppaos2* mutants behaved similar to wild type (WT) moss upon wounding. In addition, a compound accumulating upon wounding with similar accurate mass like JA, but different retention time (RT) and fragmentation pattern, was detected. By analyzing its biosynthesis additionally in the mutants of *Ppdes* and *Ppelo* we suggest that this oxylipin derives directly from the action of PpLOX1 or 2 on C20-polyunsaturated fatty acids (PUFAs).

## Materials and methods

### Moss cultures

The following genetic backgrounds were used for the non-targeted metabolomic analysis: *P. patens* (Hedw.) Bruch & Schimp: Wild type ‘Gransden’, *Ppaos1 ko 21* (in the following referred to as *Ppaos1*), *Ppaos2 ko 71* (in the following referred to as *Ppaos2*). These *P. patens* KO-mutants were generated as described by Scholz et al., 2012 and by the methods described by Frank et al., 2005.

Moss was grown on agar plates on Knop medium as described (Reski and Abel, 1985), containing for 1 L: 1000 mg Ca(NO_3_) x 4 H_2_O, 250 mg KCl, 250 mg KH_2_PO_4_, 250 mg MgSO_4_ x 7 H_2_O, 12.5 mg FeSO_4_ x 7 H_2_O (pH 5.8) and 15 g/L agar was added to the medium prior to autoclaving. Before inoculation, the inoculation moss culture was transferred to fresh Knop medium and afterwards homogenized by using an Ultra Turrax T25 basic (IKA, Stauffen, Germany) at a frequency of 19,000 min^-1^. Homogenized moss was then dropped on agar plates. Plates were inoculated with drops of about 4 weeks old homogenized liquid cultures, setting about 9 drops per plate. Plates grew at long day conditions (16 h/25°C day to 8 h/18°C night cycle) with light exposure of about 70% (55 μmol/m^2^) under normal air atmosphere. Moss was harvested after about 60 days of growth.

### Wounding moss plants

Moss wounding was performed by scraping off about 100 mg fresh gametophores from agar plate cultures and transferring them into a clean 1.5 mL reaction tube. The moss was then squashed with a plastic pestle. Subsequently, the sample was incubated under light for 30 min. The wounded sample was then frozen in liquid nitrogen. Control plants were not wounded, but incubated in 1 mL sterile tap water for 30 min under light. Three parallel cultures of each genotype and two extractions of each culture were analyzed from two independent experiments.

### Methyl-*tert*-butyl ether lipid extraction

This procedure was modified according to a method described earlier (Matyash et al., 2008). Samples (about 100 g fresh weight in 1 mL sterile tap water) were transferred to glass vials and the reaction tube in which the sample was stored was washed with 1.5 mL methanol. Afterwards, 5 mL methyl-*tert*-butyl ether (MTBE) were added, shaken vigorously and the sealed vial was shaken for 1 h at 4°C in the dark. Then, 400 μL LC-MS grade water were added to the sample, shaken vigorously and incubated for 10 min at room temperature under a fume hood. The sample was then centrifuged for 15 min at 450 *x* g and the supernatant was transferred to a new glass vial. To the remaining lower phase, 1.4 mL methanol:water, (3:2, *v/v*) and 2.6 mL MTBE were given. After vigorously shaking the sample, the vial was incubated at room temperature for 10 min under a fume hood and afterwards centrifuged for 15 min at 450 *x* g. The upper phase was then combined with the first upper phase in the new vial.

The combined non-polar upper phase was dried under streaming nitrogen and the remaining sample resolved in 1 mL methanol, then transferred to a new 1.5 mL reaction tube and dried again under streaming nitrogen. Remaining sample was resolved in 30 μL methanol, 30 μL acetonitrile, and 75 μL water. After shaking vigorously, the vial was centrifuged for 10 min at 450 x g and the supernatant was transferred to a new glass vial (storage at -20°C). The polar lower phase of the extraction described in the previous paragraph was dried under streaming nitrogen in 2 mL reaction tubes and resolved in 5 μL methanol, 10 μL acetonitrile, and 120 μL water. Afterwards, the vial was shaken vigorously and centrifuged for 10 min at 450 x g at room temperature. The supernatant was then transferred to new glass vials and stored at -20°C.

### LC-MS measurement for non-targeted metabolomics analysis

This procedure followed a method described earlier (Feussner and Feussner, 2019). For non-targeted analysis of wounded *P. patens*, non-polar and polar extractions of the samples were analyzed *via* ultra-performance liquid chromatography (UPLC, ACQUITY UPLCTM System, Waters Corporation, Milford, USA) coupled to a photo diode array detector (UPLC eLambda 800 nm, Waters Corporation, Milford, USA), followed by an orthogonal electrospray ionization time-of-flight mass spectrometer (ESI-TOF-MS, LCT Premier, Waters Corporation, Milford, USA). For analysis of the polar phase, the UPLC was equipped with an ACQUITY UPLC HSS T3 column (1.0 x 100 mm, 1.8 μm particle size, Waters Corporation, Milford, USA) held at 40°C and a flow rate of 0.2 mL/min. The following gradient program was run for the polar phase: 0 – 0.5 min 1% B, 0.5 – 3 min 1% B to 20% B, 3 – 8 min 20% B to 100% B, 8 – 10 min 100% B, 10 – 10.1 min 100% B to 1% B, 10.1 – 14 min 1% B (solvent system A: water:formic acid (100:0.1, *v/v*); B: acetonitrile:formic acid (100:0.1, *v/v*)). Analysis of the non-polar phase applied using an ACQUITY UPLC BEH C18 column (1.0 x 100 mm, 1.7 μm particle size, Waters Corporation, Milford, USA) at a temperature of 40°C and a flow rate of 0.2 mL/min. The gradient program for the non-polar phase was as follows: 0 – 0.5 min 40% B, 0.5 – 6 min 40% B to 100% B, 6 – 12 min 100% B, 12 – 12.1 min 100% B to 40% B, 12.1 – 15 min 40% B (solvent systems A and B equal to polar phase). UV/VIS data were collected in the range of 190-800 nm with a 1.2 nm resolution.

The ESI-TOF-MS was run at both positive and negative ionization mode (ESI) for all samples. In positive ESI-mode, masses from *m/z* = 85.00 – 1200.00 were scanned over a runtime of 10 min using a capillary voltage of 2700 V. In negative ESI mode, masses from m/z = 50.00 – 1200.00 were scanned over a runtime of 13 min using a capillary voltage of 2500 V. In both modes, the cone voltage was maintained at 30 V, the desolvation temperature at 350°C and the source temperature at 80°C. Data acquisition was carried out by using the MassLynx software (MassLynx V4.1 SCN779, Waters Corporation, Milford, USA) in centroid data format. Nitrogen was used as cone and desolvation gas at a flow of 30 and 800 l/h, respectively. The dynamic range enhancement mode was used for data recording. All analyses were calibrated by applying the lock spray reference compound leucine-enkephaline (Sigma-Aldrich, Deisenheim, Germany) at a concentration of 0.5 μg/mL in acetonitrile:water (50:50, *v/v*) and a flow rate of 20 μl/min.

### LC-HRMS data analysis

The raw mass spectrometry data acquired by the UPLC-HRMS analysis were preprocessed using the MarkerLynx Application Manager for MassLynx software (Waters Corporation, Milford, USA). This software tool manages peak picking and peak alignment from the raw data files to generate data matrixes. In this approach, four data matrixes are obtained: two for the metabolites of each extraction phase (polar and non-polar), analyzed in the positive and negative ESI mode. For peak detection, the following parameters were used: Initial retention time 0.30 min, final retention time 10.00 min, low mass *m/z* 50, high mass *m/z* 1200, extracted ion chromatogram window 0.03 Da. Apex track peak parameters were set to automatic.

Identification of high-quality metabolite features was conducted *via* the MarVis toolbox (Kaever et al., 2015). A metabolite feature is characterized by *m/z*, a retention time (RT) and the corresponding intensity profile received from all analyzed samples. Ranking of data applied in MarVis-Filter *via* the ANOVA ranking method. This initial ranking was combined with adjustment for multiple testing, the Benjamini-Hochberg algorithm (false discovery rate, FDR). As a threshold, only features with a FDR smaller than 1 *x* 10^-4^ were used for further data analysis. Adduct correction was performed for the four filtered data sets (non-polar phase in positive and negative ESI mode, polar phase in positive and negative ESI mode) with concern for the following adducts: [M+H]^+^, [M+Na]^+^, [M+NH_4_]^+^ (for positively charged adducts); [M-H]^-^, [M+CH_2_O_2_-H]^-^, [M+CH_2_O_2_+Na-2H]^-^ (for negatively charged adducts).

In order obtain a data set of valid metabolite features, two independent wounding experiments were analyzed by non-targeted metabolomics. Metabolite features which appeared in both data sets were used for further data analysis. All in all, 800 metabolite features remained, which were sorted into a 1D-SOM with 5 clusters, of which 4 showed a wound-depended metabolic pattern. A database search within a mass window of 0.005 Da was performed using internal databases, KEGG (Kanehisa, 2002) and AraCyc (Mueller et al., 2003).

### LC-HRMS/MS analyses

MS/MS experiments were performed *via* a UHPLC-ESI-QTOF-MS device. For UHPLC, an Agilent 1290 Infinity series UHPLC system (Agilent Technologies, Böblingen, Germany) equipped with an ACQUITY UPLC BEH C18 column (2.1 x 100 mm, 1.7 μm particle size, Waters Corporation, Milford, USA) kept at 40°C was used. Mass detection was performed with an Agilent 6540 UH Accurate-Mass-Q-TOF mass spectrometer (Agilent Technologies, Böblingen, Germany). Same solvents and gradients were used as for the non-targeted LC-MS analysis for a comparability of the two separations. The mass spectrometer was operated using an ESI source with Agilent Dual jet Stream Technology (Agilent Technologies, Böblingen, Germany) in negative ionization mode. Ionization parameters were as follows: gas temperature 300°C, gas flow 8 L/min, nebulizer pressure 35 psi, sheath gas temperature 350°C, sheath gas flow 11 L/min, VCap 3.f kV, nozzle voltage 100 V. As a collision cell, a linear hexapole collision cell with nitrogen as collision gas at various collision energies (20 eV – 40 eV) was used. For data acquisition, the Mass Hunter Workstation Acquisition Software was used (Version: B05.01.), with Mass Hunter Qualitative Analysis software as analysis tool. For structure elucidation, the fragmentation patterns of compounds were compared with MS/MS spectra deposited in the Metlin (Guijas et al., 2018) database, known from the literature (Oliw and Hamberg, 2019) or by interpretation of fragmentation rules.

### Generation of targeted KO-lines

For identifying metabolites deriving from the fatty acid 20:4 (ARA), a sample set of wounded gametophores of mutant lines for the Δ6-desaturase *Ppdes* (Girke et al., 1998) and the Δ6-elongase *Ppelo* were analyzed (Zank et al., 2002). For both genes new KO-lines were generated in the ‘Gransden’ wild-type strain. Therefore, *P. patens* protoplasts were transformed (Frank et al., 2005) with a KO-construct for the gene *Ppdes* (Pp3c5_9590) or *PpELO* (Pp3c17_7080). The KO-constructs for homologous recombination consisted of 5’ and 3’ flanking sequences of the respective genes and included a *nptII* selection cassette. Genomic DNA from gametophytic tissue was isolated using cetyl trimethylammonium bromide extraction method. PCR was performed to select transgenic lines in which either *Ppdes* or *Ppelo* was knocked out using the following primer combinations: *Ppdes*- 5’UTR: for 5’- CAAGGTGTTTCATCTCATGGTATTAG-3’/rev 5’-CCATAATAATGTGTGAGTAGTTCC-3’; 3’UTR: for 5’-CTGAGACTGTATCTTTGATATTCTTG-3’/rev 5’-ATGGCTACAGAAGAATGGATGC-3’; *Ppelo*- 5’UTR: for 5’-CAGCCACATTGTGACTATTCCCC-3’/rev 5’- CCATAATAATGTGTGAGTAGTTCC-3’; 3’UTR: for 5’-CTGAGACTGTATCTTTGATATTCTTG-3’/rev 5’-CCATCTGAAGTGCTCAGGCCG. To confirm the KO of either *Ppdes* or *Ppelo* fatty acid profiles were analyzed *via* GC and these analyses revealed the published fatty acid profiles (Girke et al., 1998; Zank et al., 2002). The lines *Ppdes28*, *Ppdes51, Ppelo35* and *Ppelo131* were selected for this work and further analyzed.

## Results

### Wounding of *P. patens* gametophores results in different chemotypes of WT and *Ppaos1* knock out lines

In order to identify wounding-related oxylipins in *P. patens* and to differentiate between an AOS-dependent as well as an AOS-independent accumulation, a non-targeted metabolite fingerprinting approach was applied. As genetic backgrounds, *P. patens* WT, *Ppaos1* and *Ppaos2* KO-mutants were used. For comparison we followed a previously optimized protocol and each genotype, unwounded control samples as well as samples harvested 0.5 hours past wounding (hpw) were analyzed by UPLC-HRMS (Stumpe et al., 2006; Stumpe et al., 2010; Scholz et al., 2012).

**Figure 1:**
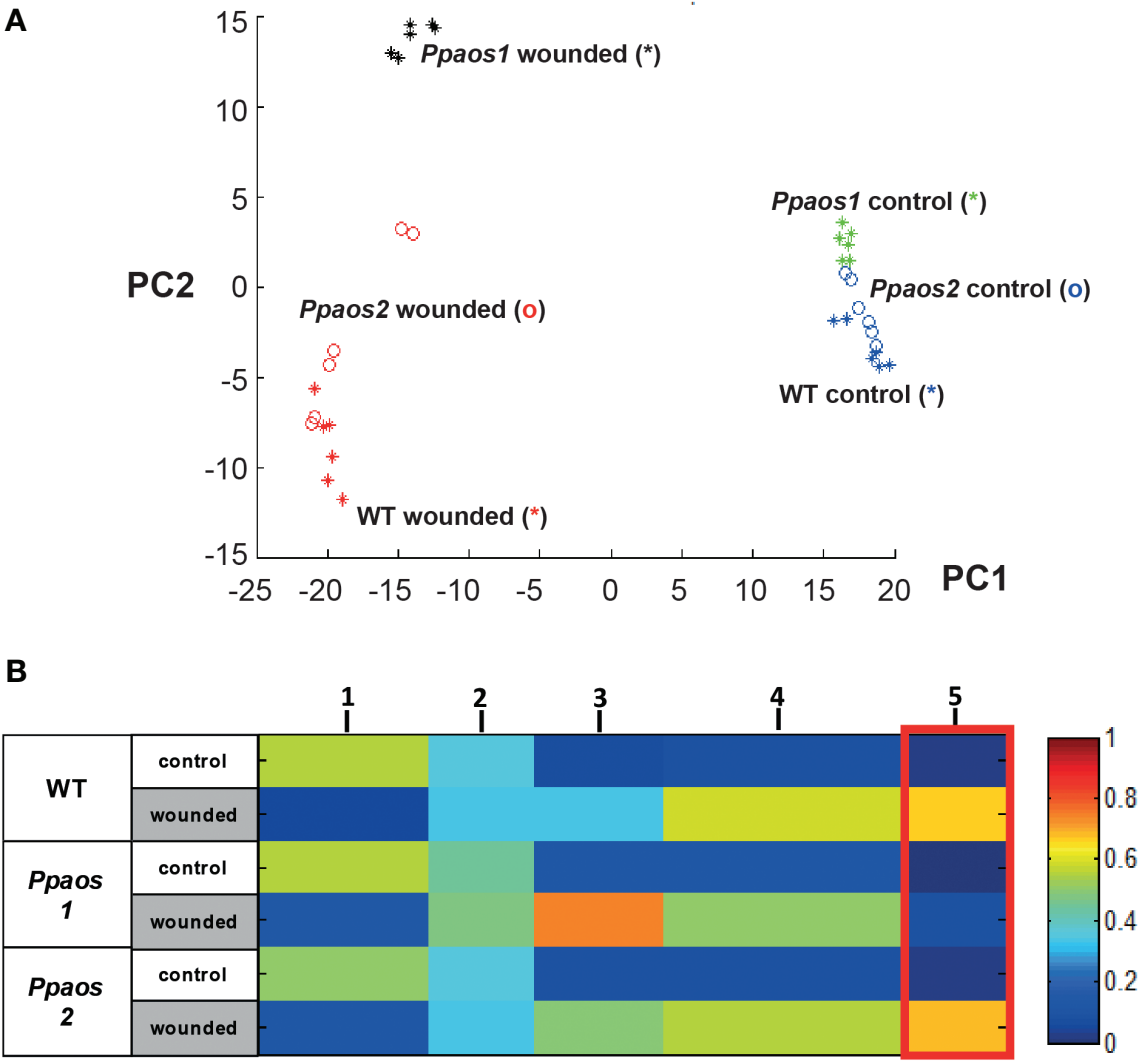
Metabolite fingerprinting analysis of a wounding experiment for *P. patens* WT, *Ppaos1* and *Ppaos2* KO-mutants. Moss cultures were grown on agar-plates at long-day conditions (16 h day/24°C to 8 h night/18°C cycle), wounded and deep frozen 30 min past wounding. After two-phase extraction, the samples were analyzed by UPLC-HR-MS. The MarVis toolbox was used for data mining (Kaever et al., 2015). 800 reliable features (FDR < 10^-4^) from two independent experiments (including data of positive and negative ionization mode of the polar and non-polar extraction phase) were used for clustering by PCA **(A)** and one-dimensional-self organizing map (1D-SOM) **(B)**. *Ppaos2* and WT moss are indistinguishable on an overall metabolite level, whereas *Ppaos1* shows a unique chemotype after wounding. Three parallel cultures of each genotype and two extractions of each culture were analyzed for the two independent experiments. The heat map colors represent average intensity values according to the color map on the right-hand side. The width of each cluster is proportional to the number of features assigned to this cluster.

Several thousand features from data matrices of the polar and the non-polar extraction phase measured both in positive and negative ionization mode were used for data mining. 800 features with a false discovery rate (FDR) < 1 x 10^-4^) occurred reproducible in two independent wounding experiments (**Supplementary Table 1**). They were used for generating a principle component analysis (PCA) (**Figure 1A**) to investigate sample-based clustering. It shows that the unwounded moss samples clustered together, implying that the different genotypes (WT, *Ppaos1* and *Ppaos2*) did not have a significant effect on the metabolome of untreated *P. paten*s. However, wounded samples are clearly separated from unwounded samples along the principle component (PC) 1 and are more widespread along the PC2, implying that the KO-lines do react differently to wounding stress. Remarkably, wild type and *Ppaos2* lines seems to react more similarly on metabolic level to the wounding stress than *Ppaos1*, indicated by a clear separation of *Ppaos1* from the other two lines.

In order to analyze the data on a global metabolite level and to identify compounds of interest, the 800 features were used for clustering by means of one-dimensional self-organizing map (1D-SOMs) using the MarVis-Cluster application of the MarVis toolbox (Kaever et al., 2009). Features with similar intensity profiles cluster together and are represented by five prototypes (**Figure 1B**). The heat map colors represent average intensity values and the width of each cluster is proportional to the number of features assigned to this cluster. The prototypes 1, 3, 4 & 5 represent metabolite features, which are either depleted (prototype 1, 182 features) or enriched (prototype 3 - 5, 505 features) upon wounding in all genotypes. Prototype 5 (marked by a red box, 107 features) summarizes features that represent compounds accumulating in all genotypes except in *Ppaos1* after wounding. As already shown by PCA (**Figure 1A**), *Ppaos2* and WT moss are indistinguishable on an overall metabolite level, whereas *Ppaos1* shows a unique chemotype after wounding.

### Oxylipins derived from roughanic, α-linolenic, linoleic, and arachidonic acid were formed after wounding

**Figure 2:**
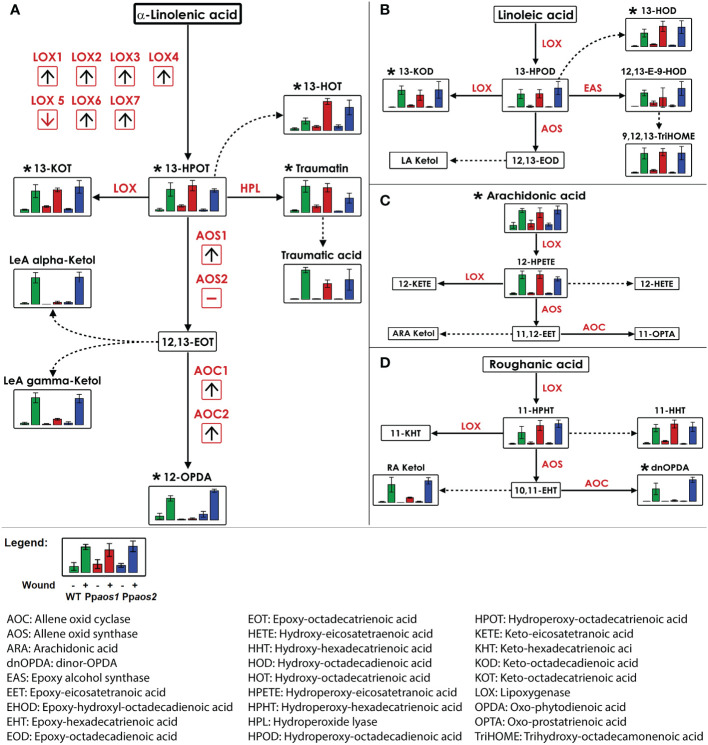
Reconstruction of LOX-pathways from metabolite fingerprinting data of a *P. patens* wounding experiment (WT, *Ppaos1*, and *Ppaos2*). Data of a non-targeted metabolome analysis acquired by UPLC-HR-MS were used for the reconstruction of LOX pathways derived from: **(A)** α-linolenic acid, **(B)** linoleic acid, **(C)** arachidonic acid, **(D)** roughanic acid. Bars show relative intensities (mean values with standard deviations) of the indicated compound of *P. patens* wild type (green), *Ppaos1* (red) and *Ppaos2* (blue) lines for control (left) and wounded gametophores (30 min past wounding, right) each. Dotted lines represent spontaneous reactions without enzymatic catalysis. Metabolites were annotated by accurate mass information. Metabolites marked with (*) were unequivocally confirmed by coelution with authentic standards and/or MS/MS experiments (**Supplementary Table 2**). Transcriptome data of a wounding experiment with *P. patens* (Hiss et al., 2014) were visualized in red boxes. Red arrows indicate the regulation of the transcript two hours post wounding by detaching leaflets from wild type moss. Pathway visualization was applied using VANTED 2.1.0 software (Rohn et al., 2012). Data represent three moss cultures per treatment. Comparable data were obtained from two independent experiments.

In order to identify the metabolites behind the features of interest and to reconstruct biosynthetic pathways, the accurate mass information of the features was used for a metabolite set enrichment analysis (Kaever et al., 2014). The pathway analysis identified 20 metabolites (represented by 46 features) from prototypes 3 - 5, which are connected to four variants of the oxylipin-pathway. These pathways start from different fatty acids: roughanic acid (RA, 16:3n-3), α-LeA, linoleic acid (LA, 18:2n-6) or arachidonic acid (ARA, 20:4n-6), respectively (**Figure 2**). The identities of 11 of the 20 metabolites were unequivocally verified by high resolution-MS/MS analyses and/or by co-elution with identical standards (**Supplementary Table 2**).

The first oxylipin formed from α-LeA is 13-hydroperoxy octadecatrienoic acid (13-HPOT). It was enriched in wounded samples of the WT and mutant lines (**Figure 2A**). Other AOS-independent metabolite patterns were observed for those metabolites deriving directly from 13-HPOT *via* LOX and hydroperoxide lyase (HPL) activities, namely 13-keto octadecatrienoic acid (13-KOT), traumatin and traumatic acid, as well as 13-hydroxy octadecatrienoic acid (13-HOT), which is spontaneously formed from 13-HPOT. 13-HOT accumulated in *Ppaos1* and *Ppaos2* even stronger than in WT samples. In contrast to that, OPDA and two variants of α-LeA-derived ketols, which are formed in direct consequence of PpAOS1 activity, were absent in the *Ppaos1* mutant, but still present in *Ppaos2* and WT samples. 12,13-epoxy octadecatrienoic acid (12,13-EOT), the direct reaction product of AOS1 and the precursor of OPDA, is very unstable and could therefore not be detected during measurements.

Furthermore, several metabolites of the LA-, ARA-, and the RA-derived LOX-pathway (**Figures 2B–D**) accumulated after wounding. The LA-derived LOX-product 13-hydroperoxy octadecadienoic acid (13-HPOD) as well as its follow-up products 13-keto octadecadienoic acid (13-KOD), 13-hydroxy octadecadienoic acid (13-HOD), 12,13-epoxy 9-hydroxy octadecadienoic acid (12,13-E-9-HOD) and 9,12,13-trihydroxy octadecaenoic acid (9,12,13-TriHOME) accumulated in all wounded genotypes. No compounds deriving from AOS1 or AOS2 catalysis were detected in the LA-pathway. ARA was the only LOX-substrate that was identified in this experiment. It showed a wound dependent accumulation in all genetic backgrounds. 12-hydroperoxy eicosatetraenoic acid (12-HPETE), the only ARA-derived oxylipin detected here, showed a similar accumulation pattern as the α-LeA-, LA-, and RA-derived hydroperoxides. The assumed ARA-derived PpAOS1 product 11-oxo prostatrienoic acid (11-OPTA) was not detectable in this experiment. Oxylipins deriving from RA were detected as 11-hydroperoxy hexadecatrienoic acid (11-HPHT) and 11-hydroxy hexadecatrienoic acid (11-HHT). With dnOPDA and a RA-derived ketol, two wounding related compounds with an AOS-dependent accumulation pattern were identified in the RA-derived pathway.

To complement the pathway reconstruction, transcriptome data from detached leaflets of *P. patens* (representing mild wounding stress) were analyzed (Hiss et al., 2014). Since experimental conditions were different, changes for the transcript levels of PpLOX1-7, PpAOS1 and 2 as well as PpAOC1 and 2, 1 and 2 hpw are only depicted as arrows showing a general trend in **Figure 2A**. Six of the seven LOXs present in *P. patens* were upregulated upon wounding. PpLOX5 was downregulated. Transcript levels of PpAOS1 rose upon wounding, while the transcription rate of PpAOS2 did not change. This is in agreement with previous findings that PpAOS1, but not PpAOS2, is involved in the wounding response of *P. patens* (Scholz et al., 2012). Additionally, the OPDA forming enzymes PpAOC1 and PpAOC2 were upregulated upon wounding, too. Together these independent transcriptome data support and complement the metabolite data from the shown oxylipin pathways.

### An extended *in silico* oxylipin database identified several AOS-dependent unknown metabolites upon wounding

Previous reports showed that the oxylipin synthesis up to OPDA and dnOPDA are present in *P. patens* while the occurrence of jasmonates is still under discussion. We wondered, if *P. patens* forms other yet unknown metabolites, which would possibly derive from OPDA or dnOPDA. These compounds could possibly fulfill similar functions as ligands of COI1, as it was described for the liverwort *M. polymorpha*, where dnOPDA has the same function as JA-Ile in flowering plants (Monte et al., 2018).

**Figure 3:**
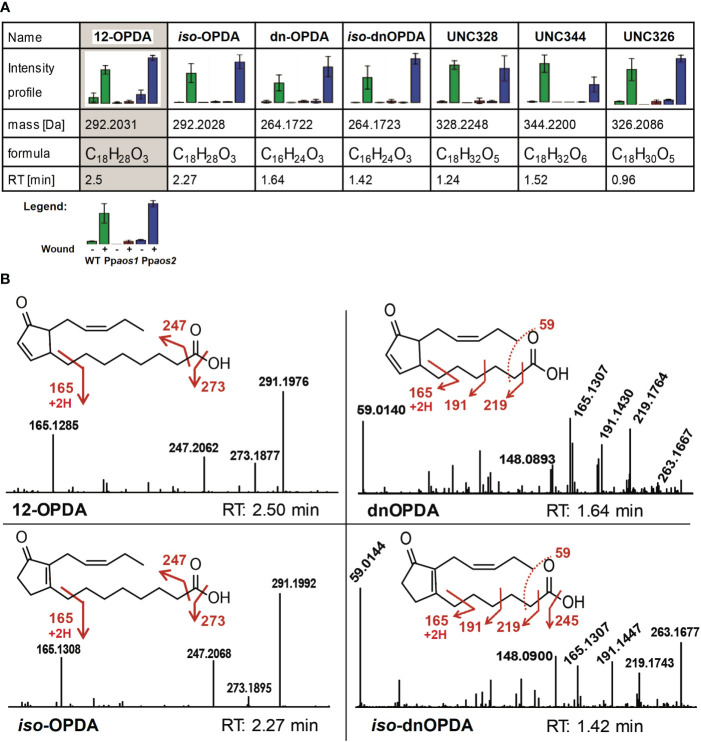
Intensity profiles and fragmentation patterns of 12-OPDA, dnOPDA and further wound-related oxylipins. **(A)** Wound-related and AOS-dependent oxylipins. Shown are intensity profile, accurate mass, retention time and the putative chemical composition derived from the accurate mass. Data was acquired using UPLC-ESI-TOF-MS (non-polar phase on a C18-reversed phase column). Of each genotype, 3 parallel cultures and of each culture 2 extractions were analyzed. Metabolic identities of features were determined using m/z, RT and intensity profiles assisted by the MarVis toolbox (Kaever et al., 2015). Bar graphs show mean values with standard deviations. Intensity pattern show: Green – P. patens WT, Red – Ppaos1, Blue – Ppaos2 (for each: left – control, right – wounded). **(B)** Some of the main compounds accumulating after wounding in *P. patens*. MS/MS fragmentation pattern and chemical structure of OPDA, *iso*-OPDA, dnOPDA and *iso*-dnOPDA are shown. Spectra were obtained by UHPLC-ESI-QTOF-MS/MS analysis in the negative ionization mode (collision energy 20 eV). Accurate m/z data can be found in **Supplementary Table 2**.

In our non-targeted metabolome data only 46 out of 505 features with a wound-accumulating pattern could be assigned to the LOX-pathways (**Figure 1B**, prototype 3-5 and **Figure 2**). Of those markers that showed an AOS1-dependent pattern, 8 out of 107 features could be assigned to 5 AOS1-dependent metabolites, like OPDA, dnOPDA and ketols (**Figure 1B**, prototype 5). The low coverage of metabolome data by searching public databases, like KEGG and MetaCyc, is a major bottleneck of the non-targeted approach, especially for the metabolome of not extensively studied organisms. This bottleneck can be addressed by implementing in-house databases into the framework of metabolite set enrichment analysis in MarVis-Pathway. We used the strategy of *in silico* database extension to increase the number of hypothetical oxylipin derivatives. Therefore, the core structures OPDA as well as dnOPDA were attached *in silico* to moieties like hydroxy, carboxy, sulfate, hexosyl, and amino acid groups, as well as combinations of those modifications. By that strategy, a database of 708 hypothetical OPDA and dnOPDA derivatives was created. Beside of OPDA and dnOPDA, 20 features representing five compounds could be identified in the data set of the non-targeted metabolome analysis by searching this *in silico* extended oxylipin database. These wound- and AOS-dependent compounds contain 18 or 16 carbon atoms (like OPDA or dnOPDA) and either an equal or higher number of oxygen and hydrogen atoms as OPDA or dnOPDA (**Figure 3A**). OPDA- or dnOPDA-like compounds linked to an amino acid or a sulfate residue were not detected.

In order to make further assumptions of the molecular structure of these compounds, LC-HRMS/MS analyses were applied to generate accurate mass fragmentation patterns. First, the identity of OPDA was confirmed. It showed three main fragments in negative ionization mode, which were assigned to loss of water [M-H_2_O-H]^-^ 273.186, loss of the carboxy group [M-CO_2_-H]^-^ 247.207, as well as the analytical OPDA fragment of *m/z* 165.125 for [M-C_7_H_10_O_2_ -H]^-^ (**Figure 3B**). These fragments were in agreement with an earlier report (Oliw and Hamberg, 2019). In a similar way the identity of dnOPDA was confirmed (**Figure 3B**). Two other wound- and AOS-dependent compounds identified in the data set are isobaric to OPDA (292.2028 Da, C_18_H_28_O_3_) and to dnOPDA (266.1725 Da, C_16_H_24_O_3_), but show earlier RTs in comparison to OPDA and dnOPDA (**Figure 3A**). The fragmentation pattern of these two compounds showed a remarkable similarity to -OPDA and dnOPDA, including the three fragments *m/z* 273.184, *m/z* 247.207 and *m/z* 165.129 of 12-OPDA, and *m/z* 219.175, *m/z* 191.144, *m/z* 165.13 and *m/z* 59.014 of dnOPDA, respectively (**Figure 3B**). In accordance with a report of Oliw and Hamberg, these two compounds were therefore assigned as *iso*-OPDA (12‐oxo‐9‐(15*Z*)‐phytodienoic acid) and *iso*-dnOPDA, respectively (Oliw and Hamberg, 2019).

The elemental composition of the three remaining unknown compounds (UNC) show 18 carbons but a higher number of hydrogen and oxygen atoms in comparison to OPDA. In detail, UNC328, shows an exact mass of 328.2248 Da and a deduced sum formula of C_18_H_32_O_5_ and is represented by 3 features (**Figure 3A**). For UNC344 (344.2200 Da, 3 features, **Figure 3A**) an elemental composition of C_18_H_32_O_6_ and for UNC326 (326.2086 Da, 3 features, **Figure 3A**) a formula of C_18_H_32_O_5_ was deduced. All three UNCs eluted earlier than OPDA on reverse phase UPLC, indicating a higher hydrophilicity than 12-OPDA because of additional functional groups, like possibly hydroxy-, oxo- or carboxy-groups.

The wound- and AOS-dependent compound UNC328 was analyzed by MS/MS fragmentation (**Supplementary Figure 1**). MS/MS analyses in negative ionization mode showed two times a loss of water (*m/z* 309.209 and *m/z* 291.198), which could suggest for additional hydroxy-groups in comparison to OPDA. The main fragments of UNC328 show an accurate ion mass of *m/z* 197.1194 and *m/z* 185.1194 with deduced elemental compositions of C_11_H_17_O_3_ and C_10_H_17_O_3_, respectively and with it a mass shift of one carbon atom. Although it shares two of its fragments (*m/z* 291.198 and *m/z* 247.207) with OPDA and *iso*-OPDA, the structure for the compound UNC328 could not be solved by MS/MS analysis.

The two other putative oxylipins (UNC344 and UNC326, **Figure 3A**) were also analyzed *via* MS/MS fragmentation. However, no suitable fragment pattern were obtained for both compounds because of low signal intensities. All in all, OPDA, *iso*-OPDA, dnOPDA, *iso*-dnOPDA, and three unknown oxylipins consisting of 18 carbon atoms and a higher number of oxygen and hydrogen atoms in comparison to OPDA were detected in wounded *P. patens* samples with an AOS-dependent intensity pattern.

### An oxylipin isobaric to JA accumulates after wounding with an AOS-independent pattern

JA-Ile is the active phytohormone in flowering plants formed after wounding. Neither JA and JA-Ile nor their direct precursors, OPC-8, OPC6 and OPC-4 were detected in any of the analyzed samples of this study. However, a compound of the same accurate ion mass as JA ([M-H]^-^ 209.1173) that accumulates upon wounding was detected in *P. patens* WT, *Ppaos1* and *Ppaos2* lines. This unknown compound (UNC210) elutes at a slightly earlier RT than the JA standard and is not diminished in *Ppaos1* and *Ppaos2* (**Supplementary Figure 2**). MS/MS-fragmentation experiments of JA and UNC210 in the negative ionization mode show for both compounds a main fragment of *m/z* 59.01, which represents the carboxylic acid moiety in JA. While *m/z* 59.01 is the only fragment generated by JA, UNC210 produces further fragments by collision induced fragmentation: *m/z* 191.106 for [M-H_2_O-H]^-^, *m/z* 147.116 for [C_11_H_15_]^-^, *m/z* 135.115 for [C_10_H_15_]^-^, *m/z* 119.085 for [C_9_H_11_]^-^, and *m/z* 93.069 for [C_7_H_9_]^-^. To investigate, if UNC210 is derived from α-linolenic acid or from different PUFAs we tested *P. patens* mutant lines of Δ6 desaturase (*Ppdes*) as well as of Δ6 elongase (*Ppelo*) for the occurrence of UNC210. *Ppdes* harbors no γ-18:3, 20:3(n-6), 20:4(n-6) and 20:5 PUFAs (Girke et al., 1998). Interestingly, in *Ppdes* lines wound induced accumulation of UNC210 is reduced. However in *Ppelo* mutants, which harbor no C20-fatty acids UNC210 is absent and does not accumulate after wounding (Zank et al., 2002). UNC210 therefore derives from C20 PUFAs and is formed independently from the activity of PpAOS1 or PpAOS2.

## Discussion

In this study, we analyzed how wounding affects gametophores of the bryophyte *P. patens* on a metabolic level and how KO-mutants of two oxylipin-forming enzymes, PpAOS1 and PpAOS2, differ on a metabolic level from *P. patens* WT.

Wounding caused significant changes in the *P. patens* metabolome, as was shown *via* a PCA (**Figure 1A**). While unwounded moss samples were virtually indistinguishable from each other regardless of the genotype, the metabolome undergoes pronounced changes after wounding in accumulating and reducing a variety of metabolites (**Figure 1B**). The *Ppaos2* mutant and the WT line react very similar to wounding. We therefore assume that those two genetic backgrounds are identical in the context of wounded gametophores and formation of oxylipins. It was determined in earlier studies that the enzyme PpAOS2 is only active on C20-PUFAs. It can form together with PpAOC2 *in vitro* the 20:4-derived oxylipin 11-OPTA, which may be an analog to the 18:3-derived OPDA (Stumpe et al., 2010; Scholz et al., 2012). Recently additional 20:4 and 20:5-derived cyclopentenones were describes from *M. polymorpha* (Kneeshaw et al., 2022). Since we did not detect any of these C20-derived cyclopentenones in our wounding experiment we assume that their formation is not part of the natural wounding response in *P. patens* gametophores. This was furthermore supported by analyzing the transcript levels of *PpAOS2* from mildly wounded gametophores (Hiss et al., 2014) which, in contrast to *PpAOS1*, did not accumulate in the damaged moss leaflets. However, the observed changes for the transcript levels were recorded for 1 and 2 hpw in contrast to the 30 minutes of wounding in this study. This might result in differences between the transcriptome data and the metabolite data from the experiments described here. In addition, it remains unclear which function 11-OPTA or any other possible C20-derived cyclopentenone fulfills in *P. patens* and under which conditions it is naturally formed in this moss.

However, we were able to identify 20 oxylipins that are formed *via* the LOX-pathway in *P. patens* after wounding. In *Ppaos1* KO-lines, 5 of these 20 wound-induced compounds were missing. This included two 18:3-derived ketols, 18:3-derived OPDA and *iso*-OPDA, one 16:3-derived ketol and 16:3-derived dnOPDA and *iso*-dnOPDA, while all other known oxylipins were not affected (**Figure 2**). Of these compounds, OPDA was one of the compounds that accumulated the strongest in wounded plants of wild type and *Ppaos2* plants. This suggests that OPDA, *iso*-OPDA, dnOPDA and *iso*-dnOPDA might play an important role in coordinating the wound response. OPDA has already been shown to suppress *P. patens* WT growth when directly applied to plate cultures (Luo et al., 2016; Monte et al., 2018). Furthermore, KO-mutants of the enzymes PpAOC1 and PpAOC2, which are involved as well as in OPDA, *iso*-OPDA, dnOPDA and *iso*-dnOPDA formation are male-sterile, hinting towards important functions of OPDA, *iso*-OPDA, dnOPDA and *iso*-dnOPDA for moss development (Stumpe et al., 2010). In addition, OPDA and dnOPDA have been described to regulate thermotolerance in *M. polymorpha* (Monte et al., 2020).

Even though dnOPDA was detected in *P. patens*, it was at relatively low signal intensities and it remains unclear what its physiological function might be in *P. patens*. The liverwort *M. polymorpha* is another bryophyte that was studied extensively regarding the formation of phytohormones. In this organism, two variants of dnOPDA were proven to interact with the COI1 receptor, which leads to activation of wounding stress response (Monte et al., 2018). We did not study metabolite-receptor interactions in this study, therefore we cannot make accurate assumptions about the role of dnOPDA in *P. patens*. However, since *P. patens* and *M. polymorpha* both represent non-vascular plants that developed as the first land-colonizing plants, we suspected some similarities in their response to the wounding stress on an oxylipin level. It is noteworthy that mosses and liverworts were probably evolutionary separated earlier than the split between vascular and non-vascular plants occurred (Clarke et al., 2011). This would allowing for further divergent evolution and could subsequently explain the difference between the two bryophytes regarding the role of dnOPDA.

The metabolic profiling *via in silico* database extension revealed several yet wound-induced UNCs to be strongly accumulated in wounded moss samples. For one substance (UNC328) the signal intensity was high enough for a MS/MS experiment (**Supplementary Figure 1**). Based on its elemental composition, two of its fragments (*m/z* 291.198 and *m/z* 247.207) that it shares with OPDA and *iso*-OPDA together with its AOS1-dependend wound-induced pattern, it is tempting to assume that it could be formed directly from OPDA (**Figure 3**). However, in case of UNC344 and UNC326 the signal intensity was not high enough for MS/MS experiments. Based on the derived elemental compositions one may assume that both substances may derive from OPDA as well. For bryophytes, it is known that OPDA itself can already act as a phytohormone related to developmental processes and biotic stress (Stumpe et al., 2010; Scholz et al., 2012; Koeduka et al., 2015; Yamamoto et al., 2015; Luo et al., 2016). In flowering plants, the modification of the OPDA-derived JA *via* amino-acid conjugation and glycosylation is described to take place (Staswick and Tiryaki, 2004; Haroth et al., 2019). Something similar might take place in *P. patens*. In addition, modification would take place at OPDA or dnOPDA as a substrate. A putative OPDA-Ile compound has already been identified before in *Arabidopsis thaliana* (Floková et al., 2016), however, here we did not detect any amino acid conjugates of either OPDA or dnOPDA in *P. patens*.

Beside *iso*-OPDA and *iso*-dnOPDA three unknown PpAOS1-dependent and wound-induced compounds were identified in this study (UNC328, UNC344, UNC326). Whether they derive directly from 12-OPDA and represent hydroxylated variants of OPDA still remains to be open. They may have even been formed independent by so far unknown enzymes or they are merely spontaneously oxidized derivates of PUFAs. Hydroxylation of phytohormones, e.g. hydroxy-JA, has been reported to occur in flowering plants for adjusting activity of these compounds (Seto et al., 2009; Caarls et al., 2017). It is noteworthy that the wounding-stress that was applied in this experiment (by grinding the plants with a pestle) could have the effect of exposing all kinds of oxylipins or PUFAs, including OPDA, to oxidizing enzymes from other cell compartments or atmospheric oxygen, resulting in uncontrolled oxidation of lipids and oxylipins. This would nevertheless mimic the effects of animals chewing or trampling on moss and could therefore represent a natural reaction to those stresses.

**Figure 4:**
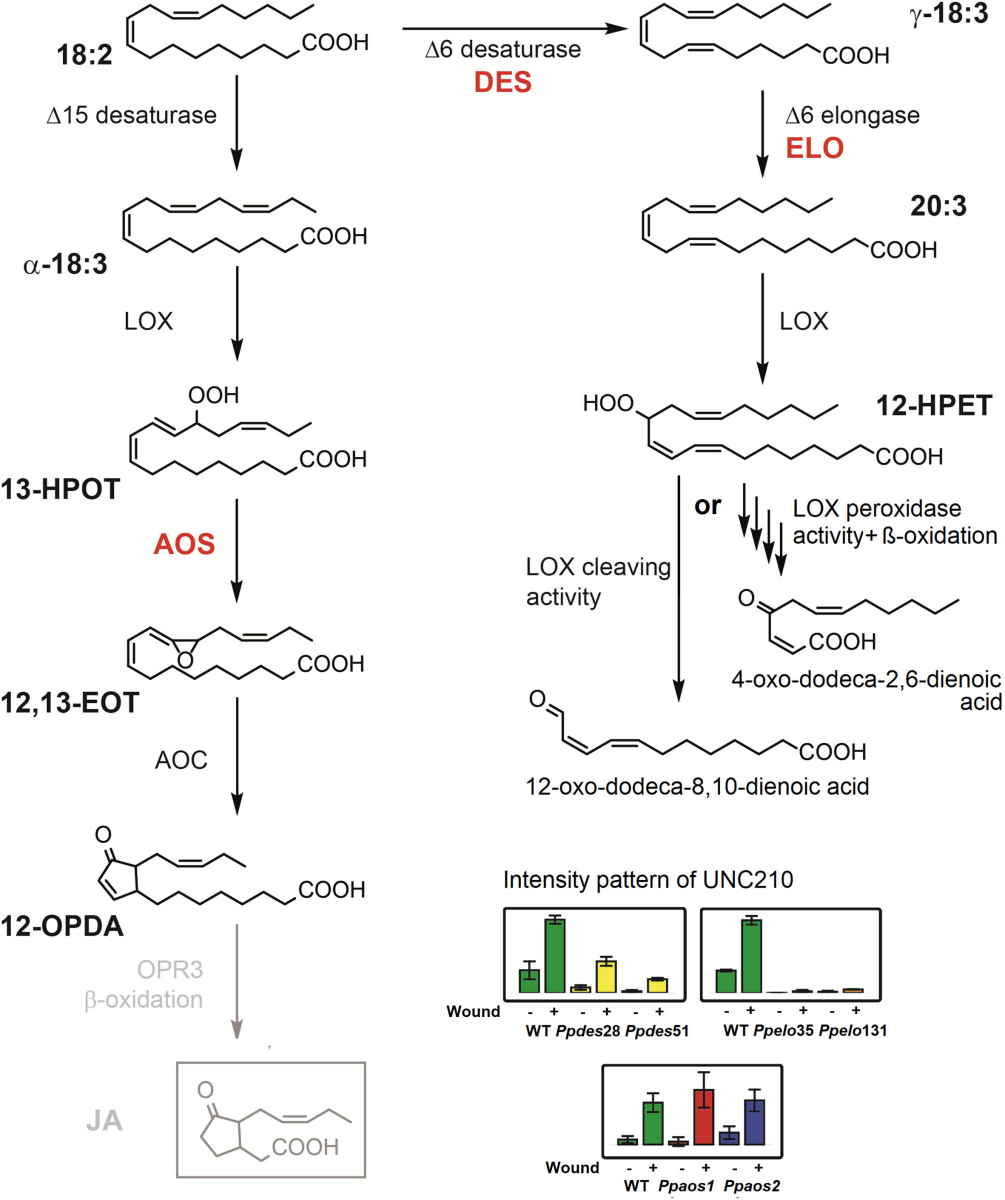
Possible pathways for the synthesis of UNC210 in response to wounding stress in *P. patens*. The compound with the mass of 210.1256 Da was detected in *P. patens*, but did not coelute with JA-standard. The intensity pattern of the compound is depicted for three separate wounding experiments (see **Figure 2**) as bar diagrams with the genetic backgrounds WT (green bars), *Ppdes*28 and 51 (Girke et al., 1998) (yellow bars), *Ppelo*35 and 131 (Zank et al., 2002) (orange bars), *Ppaos1* (red bars) and *Ppaos2* (blue bars). For each genotype, the left bar represents control samples and the right bar represents wounded samples. UNC210 accumulated strongly in wounded WT, *Ppaos1* and *Ppaos2* samples, while accumulation in wounded *Ppdes* lines was reduced. In *Ppelo* lines, the compound was not detected. Since the compound was still formed in *Ppaos1* or *Ppaos2* mutants, it cannot derive from OPDA as JA does. We therefore suggest two alternative possible structures for UNC210 that derive from the catalytic activity of PpELO and PpLOX1 or 2 on C20 PUFAs.

We furthermore confirmed former studies in which the phytohormone JA was not detected in *P. patens* at least under our experimental conditions (Stumpe et al., 2010). However, we cannot exclude its existence because it was recently detected in *M. polymorpha* (Monte et al., 2018) but the reported amounts of 1-2 pmol/g for JA in *M. polymorpha* are clearly below the detection limit of our non-targeted work flow used in this study for *P. patens*. Interestingly, we detected another wound-induced feature with the same accurate mass as JA (UNC210). In some other publications, this compound might be false-positively identified as JA, yet it differs in three key characteristics from JA: 1) It elutes at a different retention time as JA standard, 2) it is still formed in *Ppaos1* and *Ppaos2* KO-mutants, and 3) it requires the presence of C20 fatty acids to be formed in *P. patens*. Therefore, it is very likely that UNC210 derives from the activity of PpLOX1 or 2 which have been described as arachidonate 12-LOXs (Anterola et al., 2009). UNC210 may then either directly derive from the 12-HPETE-cleaving activity or it is formed by the peroxidase activity of these enzymes and following three rounds of β-oxidation (**Figure 4**) (Senger et al., 2005). A similar reaction sequence to this latter possibility has been described before to occur in sunflower cotyledons and further experiments are needed to determine the exact biochemical pathway for the synthesis of UNC210 (Gerhardt et al., 2005). Since UNC210 shares the prominent *m/z* 59.013 fragment with JA and dnOPDA (**Figure 3B** and **Supplementary Figure 2**) which corresponds to a loss of an acetate moiety, it may be more likely that UNC210 is directly formed by the activity of PpLOX1 or 2 (**Figure 4**, left pathway on the right). UNC210, or another similar compound, might also be present in other plants that are able to synthesize PUFAs with 20 carbons chain length. However, further studies would have to be conducted in the future to test this theory.

## Conclusion

The COI1 receptor in plants does not necessarily require the presence of JA-Ile to interact with, as was described for the bryophyte *M. polymorpha* (Monte et al., 2018). *P. patens* appears to possess similar features, with one or more variants of the COI1 receptor present in the genome, but its ligand needs still to be discovered (Han, 2017). Our non-targeted metabolomics study did reveal several known oxylipins (OPDA, dnOPDA *iso-*OPDA, *iso-*dnOPDA), several additional compounds to accumulate after wounding (UNC328, UNC344, UNC326) and an compound being isobaric to JA (UNC210) that could fulfill this function in *P. patens*. Therefore, the findings of this study can serve as foundation for future research in determining the structure of the identified UNCs. Considering the very low amounts of JA that accumulate in *M. polymorpha* upon wounding optimizing the sensitivity of the workflow for the detection of jasmonates in *P. patens* may be another option. All in all, this may lead to the identification of the ligand of the COI1-receptor in *P. patens* and it will be interesting to see whether it is conserved in bryophytes.

## Data availability statement

The original contributions presented in the study are included in the article/**Supplementary Material**. Further inquiries can be directed to the corresponding author.

## Author contributions

HR, KF, EH, and IF conceived and designed the experiments. HR, EH, and KF performed the experiments. HR, EH, KF, and IF analyzed and discussed the data, HR, KF, and IF wrote the article. All authors contributed to the article and approved the submitted version.

## Glossary

**Table:**

1D-SOM	one-dimensional-self organizing map
AOC	allene oxide cyclase
AOS	allene oxide synthase
ARA	arachidonic acid
E-HOD	epoxy hydroxy octadecadienoic acid
FDR	false discovery rate
HHT	hydroxy hexadecatrienoic acid
HOD	hydroxy octadecadienoic acid
HOT	hydroxy octadecatrienoic acid
HPETE	hydroperoxy eicosatetraenoic acid
HPHT	hydroperoxy hexadecatrienoic acid
HPOD	hydroperoxy octadecatrienoic acid
HPOT	hydroperoxy octadecatrienoic acid
hpw	hours past wounding
JA	jasmonic acid
JA-Ile	jasmonic acid isoleucine conjugate
KOT	keto octadecatrienoic acid
KO	knock-out
LA	linoleic acid
α-LeA	α-linolenic acid
LOX	lipoxygenase
MTBE	methyl-*tert*-butyl ether
OPDA	12-oxo phytodienoic acid
*iso*-OPDA	12‐oxo‐9‐(15*Z*)‐phytodienoic acid
dnOPDA	*dinor*-OPDA
OPR3	OPDA-reductase isoform 3
OPTA	oxo prostatrienoic acid
PC	principle component
PCA	principle component analysis
PUFA	polyunsaturated fatty acid
RA	roughanic acid
RT	retention time
TriHOME	trihydroxy octadecaenoic acid
UFA	unsaturated fatty acid
UNC	unknown compound
UPLC-HR-MS	ultra-performance-liquid-chromatography high-resolution mass spectrometry
WT	wild type
