# Supplementary material for: A non-targeted metabolomics analysis identifies wound-induced oxylipins in Physcomitrium patens
Source: Front Plant Sci. 2023 Jan 10;13:1085915. doi: 10.3389/fpls.2022.1085915 (PMC9871578; doi:10.3389/fpls.2022.1085915)
Supplement: Supplementary file 5 [file DataSheet_1.pdf]

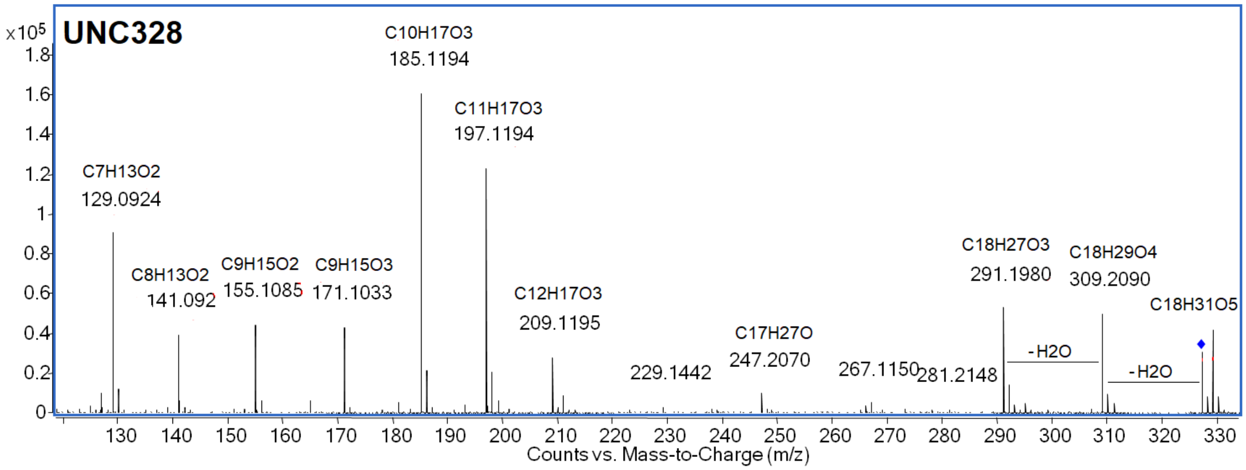

Supplementary Figure 1. HR-MS/MS fragmentation pattern of UNC328. Spectrum was obtained by UHPLC-ESI-QTOF-MS/MS analysis in the negative ionization mode (collision energy 20 eV).
