# Supplementary material for: A non-targeted metabolomics analysis identifies wound-induced oxylipins in Physcomitrium patens
Source: Front Plant Sci. 2023 Jan 10;13:1085915. doi: 10.3389/fpls.2022.1085915 (PMC9871578; doi:10.3389/fpls.2022.1085915)
Supplement: Supplementary file 6 [file DataSheet_2.pdf]

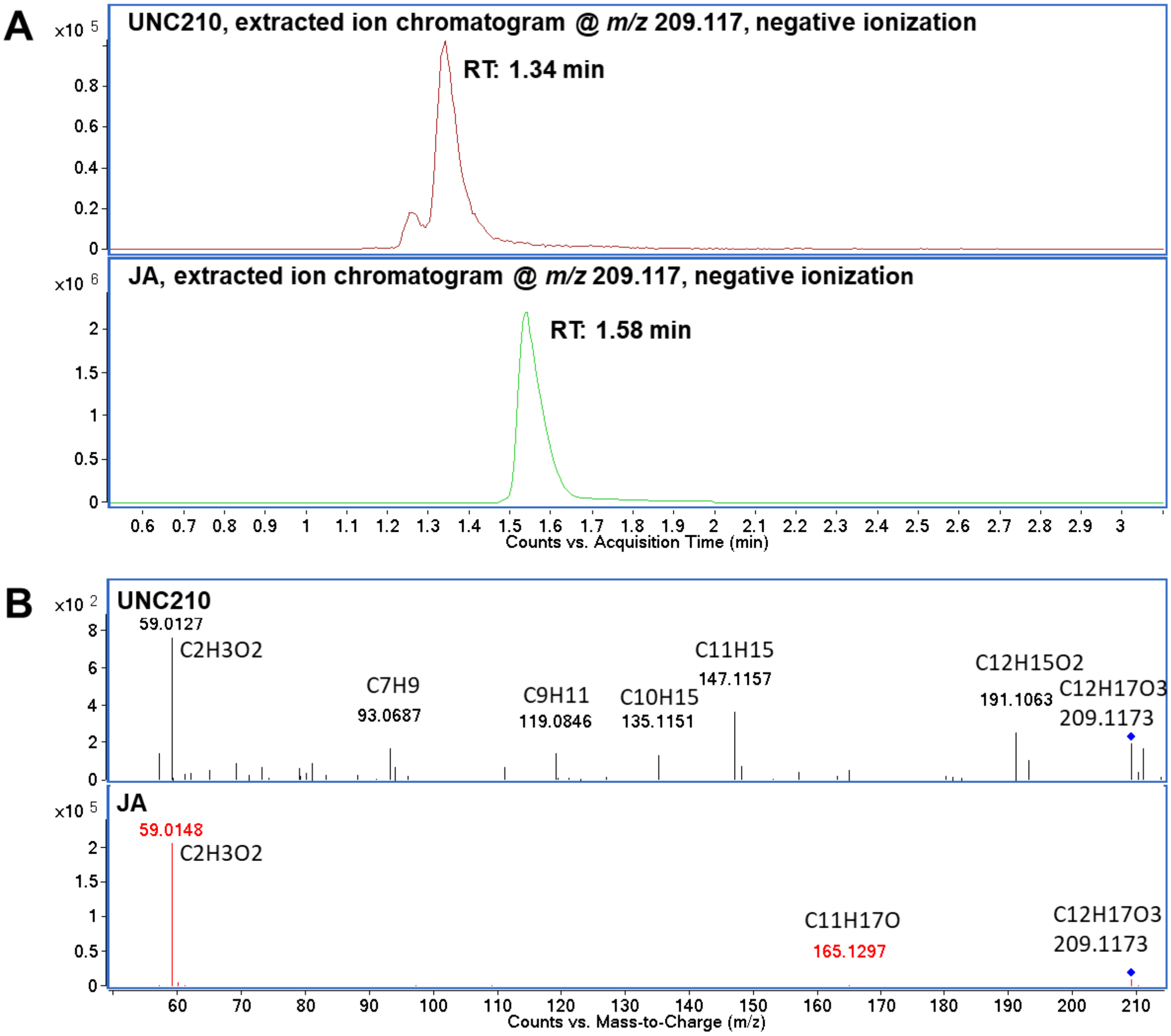

Supplementary Figure 2. Extracted ion chromatograms (A) and HR-MS/MS fragmentation pattern (B) of JA and the isobaric compound UNC210. Spectra were obtained by UHPLC-ESI-QTOF-MS/MS analysis in the negative ionization mode with the following collision energies: 10 eV (UNC210) 12 eV (JA).
